# [Retracted] Astragaloside IV inhibits platelet‑derived growth factor‑BB‑stimulated proliferation and migration of vascular smooth muscle cells via the inhibition of p38 MAPK signaling

**DOI:** 10.3892/etm.2023.12250

**Published:** 2023-10-16

**Authors:** Zhuo Chen, Ying Cai, Wenliang Zhang, Xinzhou Liu, Suixin Liu

Exp Ther Med 8:1253–1258, 2014; 10.3892/etm.2014.1905

Following the publication of this paper, it was drawn to the Editors’ attention by a concerned reader that the western blotting data shown in Fig. 2, 3 and 5, and the Transwell migration assay data in Fig. 4, were strikingly similar to data appearing in different form in other articles written by different authors at different research institutes that had either already been published elsewhere prior to the submission of this paper to *Experimental and Therapeutic Medicine*, or were under consideration for publication at around the same time. In view of the fact that these data had already apparently been published previously, the Editor of *Experimental and Therapeutic Medicine* has decided that this paper should be retracted from the Journal. The authors were asked for an explanation to account for these concerns, but the Editorial Office did not receive a reply. The Editor apologizes to the readership for any inconvenience caused.

